# Supplementary material for: The interactome of intact mitochondria by cross-linking mass spectrometry provides evidence for coexisting respiratory supercomplexes
Source: Mol Cell Proteomics. 2017 Dec 8;17(2):216–32. doi: 10.1074/mcp.RA117.000470 (PMC5795388; doi:10.1074/mcp.RA117.000470)
Supplement: Supplemental Data [file supp_RA117.000470_133922_0_supp_23978_fzffwf.zip › spectra_annotation/mito_spectra_annotation/79-1-11-1-5-1.pdf]

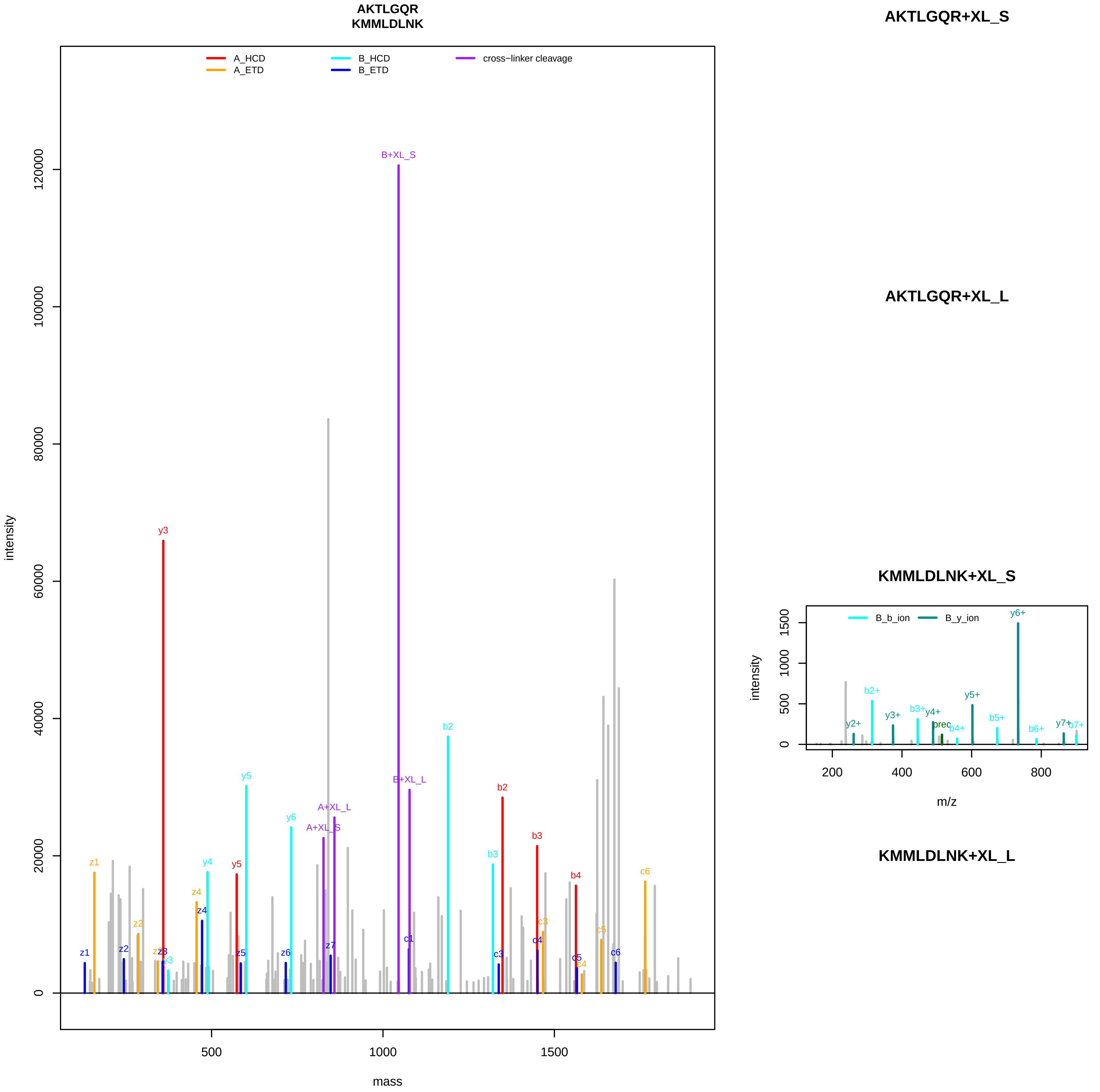
AKTLGQR
KMMLDLNK
A_HCD
A_ETD
B_HCD
B_ETD
cross−linker cleavage
intensity
mass
0
20000
40000
60000
80000
100000
120000
500
1000
1500
B+XL_S
B+XL_L
A+XL_L
A+XL_S
y3
y4
y5
y6
b2
b3
b4
c1
c3
c4
c5
c6
z1
z2
z4
z5
z6
z7
AKTLGQR+XL_S
AKTLGQR+XL_L
KMMLDLNK+XL_S
B_b_ion
B_y_ion
intensity
m/z
0
500
1000
1500
200
400
600
800
y2+
y3+
y4+
y5+
y6+
y7+
b2+
b3+
b4+
b5+
b6+
b7+
prec
KMMLDLNK+XL_L
